# Evolution of Si Crystallographic Planes-Etching of Square and Circle Patterns in 25 wt % TMAH

**DOI:** 10.3390/mi10020102

**Published:** 2019-01-31

**Authors:** Milče M. Smiljanić, Žarko Lazić, Branislav Radjenović, Marija Radmilović-Radjenović, Vesna Jović

**Affiliations:** 1 Institute of Chemistry, Technology and Metallurgy-Centre of Microelectronic Technologies (IHTM-CMT), University of Belgrade, Njegoševa 12, 11000 Belgrade, Serbia; zlazic@nanosys.ihtm.bg.ac.rs (Z.L.); vjovic@nanosys.ihtm.bg.ac.rs (V.J.); 2 Institute of Physics, University of Belgrade, Pregrevica 118, 11080 Belgrade, Serbia; bradjeno@ipb.ac.rs (B.R.); marija@ipb.ac.rs (M.R.-R.)

**Keywords:** tetramethylammonium hydroxide (TMAH), wet etching, silicon, 3D simulation, level-set method

## Abstract

Squares and circles are basic patterns for most mask designs of silicon microdevices. Evolution of etched Si crystallographic planes defined by square and circle patterns in the masking layer is presented and analyzed in this paper. The sides of square patterns in the masking layer are designed along predetermined <n10> crystallographic directions. Etching of a (100) silicon substrate is performed in 25 wt % tetramethylammonium hydroxide (TMAH) water solution at the temperature of 80 °C. Additionally, this paper presents three-dimensional (3D) simulations of the profile evolution during silicon etching of designed patterns based on the level-set method. We analyzed etching of designed patterns in the shape of square and circle islands. The crystallographic planes that appear during etching of 3D structures in the experiment and simulated etching profiles are determined. A good agreement between dominant crystallographic planes through experiments and simulations is obtained. The etch rates of dominant exposed crystallographic planes are also analytically calculated.

## 1. Introduction

Anisotropic wet etching of a (100) silicon substrate in 25 wt % tetramethylammonium hydroxide (TMAH) water solution is a well-known process [1,2,3,4,5,6,7,8,9,10,11]. Most of the results have been obtained through the analysis of the etching square or rectangular patterns in the masking layer with sides along <110> crystallographic direction. During etching of these mesa structures, a severe convex corner undercutting appears. The convex corner compensation techniques for the TMAH water solutions etching are developed for the patterns with sides along <110> crystallographic directions [12,13,14,15,16,17,18,19,20,21,22]. Additionally, etched circular patterns were compared for pure and surfactant added 25 wt % TMAH water solution [23]. When a (100) silicon substrate is etched through the aperture in the masking layer that only has concave angles, the structure shape will be defined after a sufficiently long etching time with the slowest etching crystallographic planes {111}. Appearance of {111} is inevitable and cannot be eliminated.

Previous studies of silicon wet etching processes [1,2,3,4,5,6,7,8,9,10,11,12,13,14,15,16,17,18,19,20,21,22,23,24,25,26,27,28,29,30,31,32] were conducted using various etching solutions of KOH and TMAH at different temperatures and silicon wafers of various crystallographic orientations. Etching of a (100) silicon in the KOH water solution using circular mask patterns, as well as square, rectangular and octagonal mask patterns with sides along different crystallographic directions was explored in References [2,26,27,28,29,30,31,32]. Etching of circular and square patterns with sides along <100> crystallographic directions was analyzed in References [26,27,28]. Etching of squares with sides along <210> and <310> crystallographic directions was studied to develop triangular corner compensation structure [31]. Etching of octagonal patterns with sides along <210>, <310> and <410> crystallographic directions was discussed in References [28,29] for KOH, and in Reference [9] for TMAH water solutions. Additionally, etching of squares [32] and rectangles [30] was analyzed as their side directions change angle with <110> crystallographic direction. A special case study considered changing of the obtuse angle with an increment of 2° [24]. In this paper, we explore etching of square patterns that are designed along predetermined <n10> crystallographic directions (0 ≤ n < 10) and circle patterns in the masking layer on a (100) silicon. Squares (rectangles) and circles are basic patterns for a great majority of mask designs in micromachining of membranes, bosses, convex corner compensations, etc. We analyze etching of square and circle islands in the masking layer using both experiments and simulations, and provide a comprehensive insight into the evolution of patterns for different crystallographic directions. Etched silicon structures are limited by various planes during etching in 25 wt % TMAH water solution at a temperature of 80 °C. Because of the differences in etch rates, some planes will appear, while others will disappear during etching [1,2,3,4,5,6,7,8,9,10,11,12,13,14,15,16,17,18,19,20,21,22,23,24]. We performed our experiments for the etching depths of up to 300 µm.

Our aim is to observe and analyze the appearance of various crystallographic planes and verify agreement of simulation with experimental results. Knowing evolution of crystallographic planes during etching is necessary for the successful mask design of silicon microdevices. Simulated etching profiles are obtained by the level-set (LS) method [33,34,35,36,37,38,39,40]. This method for evolving interfaces belongs to the geometric type of methods, and it is specially designed for profiles that can develop sharp corners, change of topology and undergo orders of magnitude changes in speed. All simulations are performed using a three-dimensional (3D) anisotropic etching simulator based on the sparse field method for solving the level-set equations, described in our previous publications [13,34,35,36,37]. Pictures of the simulated etching profiles are rendered with the Paraview visualization package [41]. The scanning electron microscopy (SEM) micrographs for several subsequent etch depths and simulated etching profiles are presented to demonstrate evolution of all exposed crystallographic planes. Additionally, we derive relation between parameters to calculate etch rates of the exposed planes. The etch rates are determined by measuring change of the side *a* of square island in the masking layer with time.

## 2. Experimental Setup

We used phosphorus-doped {100} oriented 3″ silicon wafers (Wacker, Burghausen, Germany) with mirror-like single side polished surfaces and 1–5 Ωcm resistivity. Anisotropic etching was performed in pure TMAH 25 wt % water solution (Merck, Darmstadt, Germany). The etching temperature was 80 °C. Wafers were standard Piranha and RCA cleaned and covered with SiO_2_ thermally grown at 1100 °C in an oxygen ambient saturated with water vapour (at least 1 μm thick). Cleaning of Si wafers before oxidation was accomplished by using freshly-prepared mixture of concentrated sulfuric acid (H_2_SO_4_, 95–98%) and hydrogen peroxide (H_2_O_2_, 30%). Mixture had volume ratio 3:1 (H_2_SO_4_:H_2_O_2_). RCA processing steps used a mixture of ammonium hydroxide (NH_4_OH, 29%), hydrogen peroxide and water (1:1:5) and a mixture of hydrochloric acid (HCl, 37%), hydrogen peroxide and water (1:1:6). SiO_2_ was etched in buffered hydrofluoric acid (BHF) in a photolithographic process in order to define square patterns along predetermined crystallographic directions and circle patterns. BHF solution consisted of 7 parts by volume of ammonium fluoride (NH_4_F, 40%) and 7 parts by volume of hydrofluoric acid (HF, about 50%.) Again, wafers were subjected to standard cleaning procedure and were dipped before etching for 30 s in HF (10%) to remove native SiO_2_ followed by rinsing in deionized water. Etching of the whole 3″ wafer was carried out in a thermostated glass vessel containing around 0.8 dm^3^ of the solution with electronic temperature controller stabilizing temperature within ± 0.5 °C. The vessel was on the top of a hot plate and closed with a Teflon lid that included a water-cooled condenser to minimize evaporation during etching. The wafer was oriented vertically in a Teflon basket inside the glass vessel. Throughout the process, the solution was electromagnetically stirred with a velocity of 300 rpm. After reaching the desired depth, the wafer was rinsed in deionized water and dried with nitrogen.

## 3. Discussion and Results

### 3.1. Square Islands in the Masking Layer with Sides Along <n10> Crystallographic Directions

Various 3D shapes were obtained by etching of square islands in the masking layer with sides along <n10> crystallographic directions. At the beginning of etching, sides of 3D structure aligned to <n10> direction belonging to {n11} crystallographic planes. The convex corners were undercut by fast etching crystallographic planes. As etching continued, for almost all square patterns, fast etching crystallographic planes dominated. Convex corners of squares differed from case to case, and their shapes changed with etching depth. Some of them were defined with rugged, others were defined with smooth crystallographic planes.

**Figure 1:**
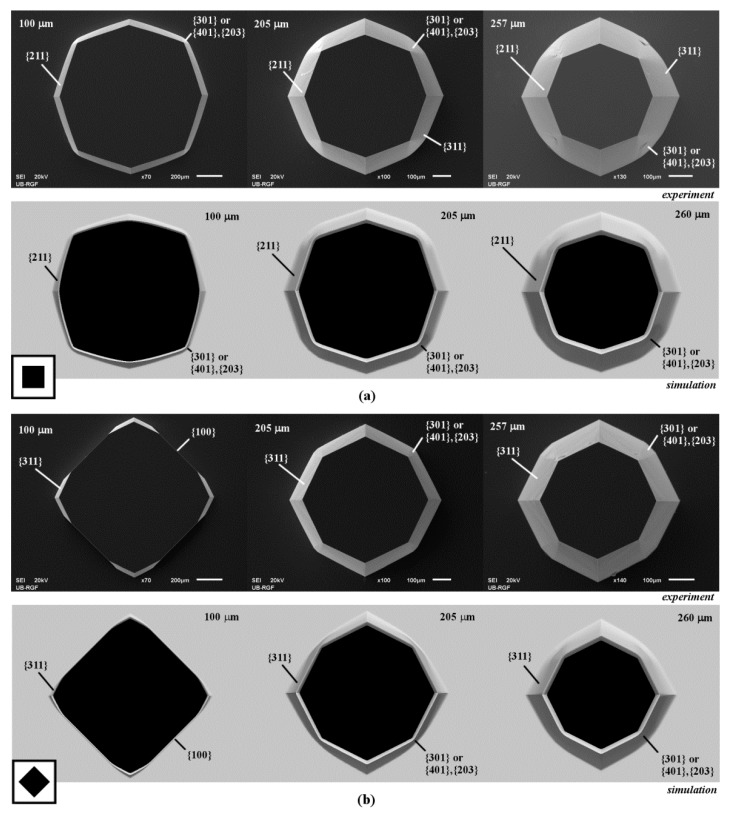
Shematic mask patterns, scanning electron microscopy (SEM) micrographs and simulated etching profiles of the etched square island with sides along: (**a**) <110> directions; (**b**) <100> directions. In the experiment the depths of etching were 100, 205 and 257 µm. In the simulation the depths of etching were 100, 205 and 260 µm.

**Figure 2:**
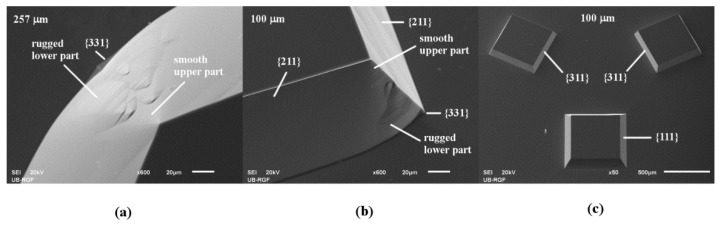
SEM micrographs: (**a**) Enlarged detail of the smooth upper and rugged lower part of undercut convex corner of etched square island with sides along <110> directions; (**b**) Convex corners undercut asymmetrically by the smooth upper and rugged lower part in the case of etched square island with sides along <210> directions; (**c**) Etched silicon structures from square island with sides along <110> directions with convex corner compensation and two symmetrical square islands with sides along <310> directions in the masking layer.

In the case of square island with sides along <110> directions, the slowest etching planes {111} defined sidewalls of silicon structure. There was a severe convex corner undercutting. Convex corner is defined by the rugged planes of {m0n} families and smooth planes of {311} and {211} families [13,24,42], as shown in Figure 1a. Observing etched shapes obtained in the experiments, we conjectured that the rugged planes of {m0n} families belong to the {301} or {401} families because the average angles *γ_m0n_* between {m0n} planes and (100) plane are close to 71° and 75°. We also noticed in SEM micrographs that most of the {m0n} planes consist of smooth upper and rugged lower part, as shown in Figure 2a. In the simulated etching profiles there are two different planes instead of one from the {m0n} family. Average angles of intersections of these {m0n} planes and (100) silicon plane are close to 75° and 55.8°. We assume that they belong to the {401} and {203} families, where the {401} plane is closer to the masking layer and the {203} plane to the etched bottom. The appearance of two planes in the simulation is likely to be related to the smooth upper and rugged lower parts of the {m0n} planes in the SEM micrographs. Additionally, at the bottom of the {m0n} planes, there are two almost imperceptible symmetrical planes, as shown in Figure 2a. We assume that they belong to the {331} family. As etching continues, the plane of {111} family on the sidewall of structure disappears and two smooth planes of {211} family from the nearby convex corner define new convex corner, as shown in Figure 1a. Two smooth planes of {211} family are slightly undercut by planes that are hard to determine. Further etching will not change the silicon structure defined by the new convex corners and undercut convex corners.

In the case of square with sides along <100> directions, etching planes of the {100} family define sidewalls of the silicon structure, as shown in Figure 1b. As in the previous case, there is severe undercutting of the convex corner but only by the smooth planes of {311} family, as shown in Figure 1b. As etching continues, the {100} plane on the sidewall of structure disappears and two planes of {311} family from the nearby convex corners adjoin. At their cross section, new fast etching rugged planes from presumed {301} (or {401}–{203}) and {331} families appear, as shown in Figure 1b. The new shape of the silicon structure with eight convex corners sustains during the further etching. Similar shape was obtained in Reference [9] when etching with an octagonal mask in the pure TMAH 25 wt % water solution. The authors observed that the planes of {311} family are inclined only to the surface, while the planes of {331} family are at the bottom.

**Figure 3:**
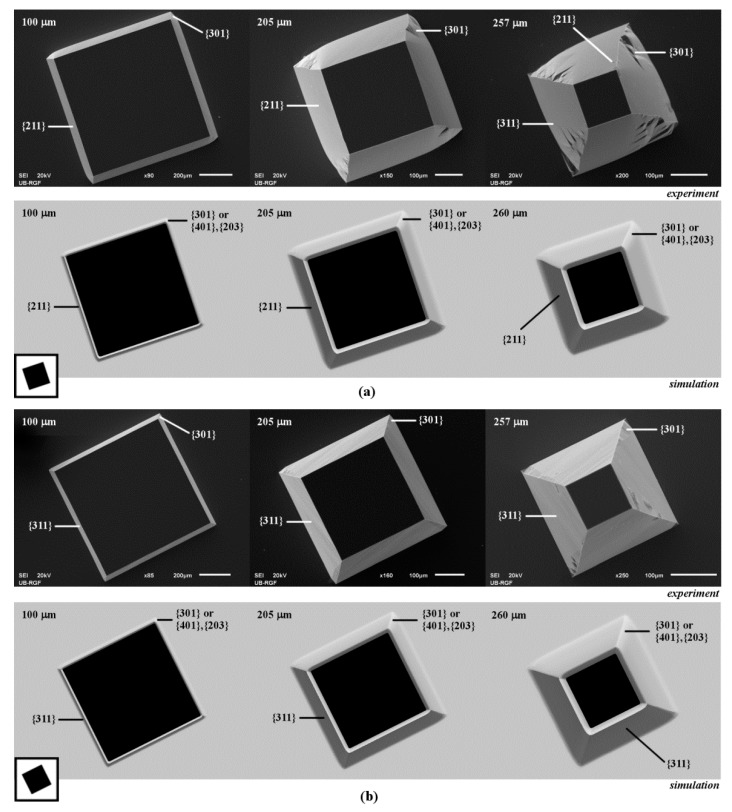
Schematic mask patterns, SEM micrographs and simulated etching profiles of the etched square island with sides along: (**a**) <210> directions; (**b**) <310> directions. In the experiment the depths of etching were 100, 205 and 257 µm. In the simulation the depths of etching were 100, 205 and 260 µm.

3D shapes obtained by etching of square islands with sides along <210> and <310> crystallographic directions are very similar, as shown in Figure 3. If the sides are along <210> directions, at the beginning of etching obtained shape is a truncated pyramid with sides defined by the planes of {211} family. In Reference [9], the planes of {211} family are inclined to the surface, and the planes of {221} family are at the bottom. These planes were observed at the etched octagonal mask shape with sides along <210> crystallographic directions in pure TMAH 25 wt % water solution. Convex corner undercutting appears, as shown in Figure 3a. Convex corner is defined by the rugged planes of {301} (or {401}–{203}) and {331} families and the smooth planes of {211} family, as shown in Figure 2b. This asymmetrical undercutting is less destructive than in the case of square patterns with sides along <110> and <100> directions due to smaller differences of etch rates [13]. After a sufficiently long etching time, the shape will be changed into truncated pyramid defined by {311} planes. Convex corner’s shape will not be changed. If the sides are along <310> directions, the pyramidal shape with sides defined by the planes of {311} family is formed from the beginning of etching. In Reference [9], the planes from {311} family are inclined to the surface, and planes of {331} family are at the bottom in the case of octagone sides along <310> crystallographic directions. As in the previous case, there is also asymmetrical undercutting of the convex corner by the rugged planes of {301} family (or {401}–{203} families), as shown in Figure 3b. Additionally, at the bottom, there is almost imperceptible plane from the presumed {331} family. This 3D shape will not be changed during further etching.

**Figure 4:**
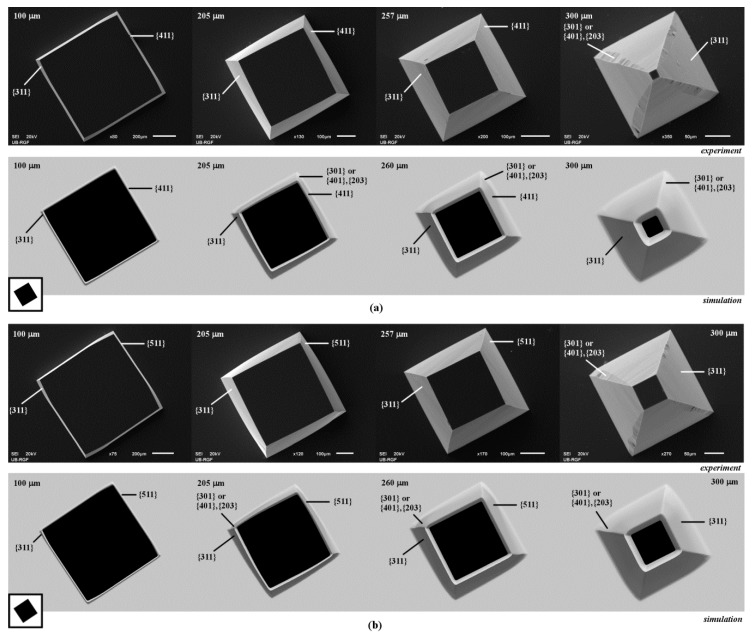
Schematic mask patterns, SEM micrographs and simulated etching profiles of the etched square island with sides along: (**a**) <410> directions; (**b**) <510> directions. In the experiment the depths of etching were 100, 205, 257 and 300 µm. In the simulation the depths of etching were 100, 205, 260 and 300 µm.

**Figure 5:**
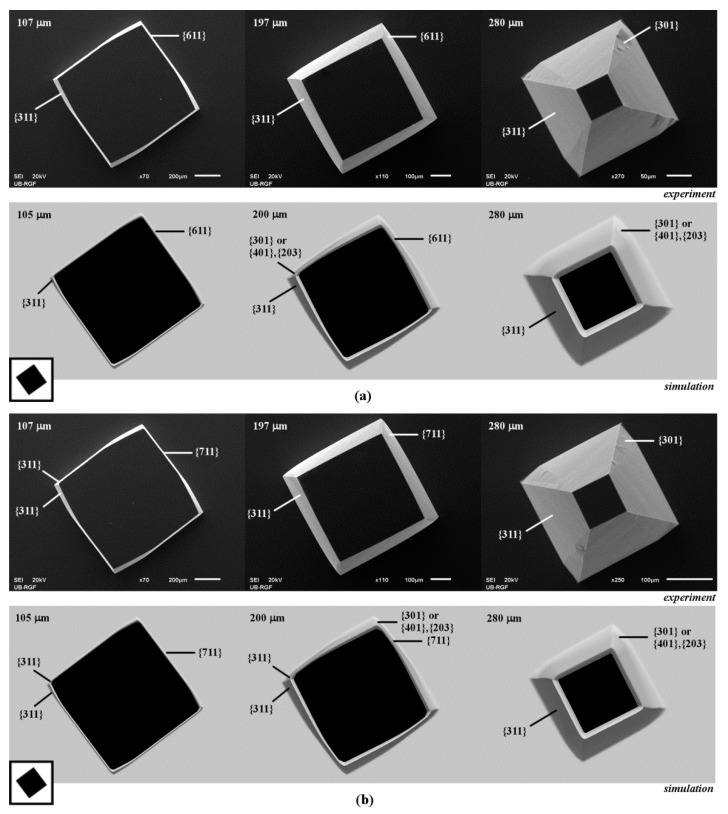
Schematic mask patterns, SEM micrographs and simulated etching profiles of the etched square island with sides along: (**a**) <610> directions; (**b**) <710> directions. In the experiment the depths of etching were 107, 197 and 280 µm. In the simulation the depths of etching were 105, 200 and 280 µm.

We observed that the 3D shapes obtained in the cases of etching of square islands with sides along <410>, <510> and <610> crystallographic directions are also very similar, as shown in Figure 4 and Figure 5a. In the case of the square with sides along <410> directions, at the beginning of etching pyramidal shape with sides defined by planes of {411} family is obtained. Same planes were also noticed in Reference [9] for the octagone with sides along <410> crystallographic directions. Convex corner undercutting appears again, as shown in Figure 4a. Convex corner is defined by the smooth planes of {311} and {411} families. Neither planes of {301} nor the planes of {401}–{203} families appear. As etching continues, the {411} plane on the sidewall of pyramid disappears and smooth {311} plane from the nearby convex corner starts to dominate, as shown in Figure 4a. The shape will be changed into truncated pyramid after a sufficiently long etching time as in the case of square island with sides along <310> crystallographic directions. Evolution of the etched silicon structure will be the same in the cases of square islands with sides along <510> and <610> crystallographic directions, as shown in Figure 4b and Figure 5a. At the beginning, the pyramid sidewalls will be the planes of {511} and {611} families, respectively. Convex corners are defined by the smooth planes of {311} and {511} or {311} and {611} families, as shown in Figure 4b and Figure 5a. As in the previous case, the {311} planes dominate after a sufficiently long etching time. Again, pyramidal shapes are obtained as in the case of square island with sides along <310> crystallographic directions. It can be noticed that planes of {411}, {511} and {611} families are not as smooth as planes of {211} and {311} families. It looks like that they consist of consecutive facets of negligible areas.

**Figure 6:**
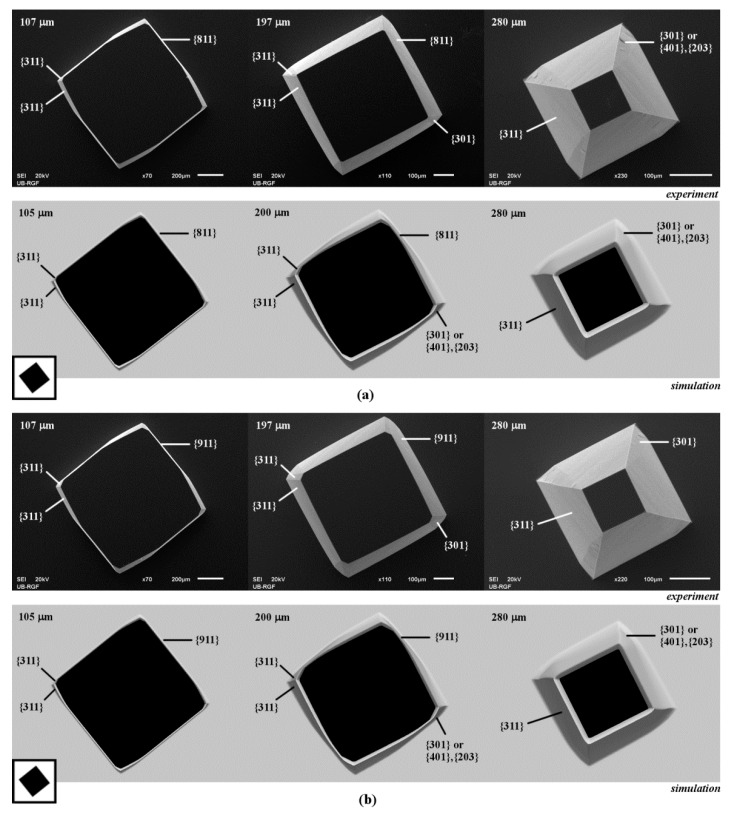
Schematic mask patterns, SEM micrographs and simulated etching profiles of the etched square island with sides along: (**a**) <810> directions; (**b**) <910> directions. In the experiment the depths of etching were 107, 197 and 280 µm. In the simulation the depths of etching were 105, 200 and 280 µm.

Other similarities can be noticed when observing the 3D shapes obtained by etching of the square islands with sides along <710>, <810> and <910> crystallographic directions, as shown in Figure 5b and Figure 6. In the case of the square with sides along <710> directions, the pyramidal shape with sides defined by the planes of {711} family is obtained at the beginning of etching, as shown in Figure 5b. Asymmetrical convex corner’s undercut is done by two smooth {311} planes, as in the case of square island with sides along <100> directions, as shown in Figure 1b. At the cross section of the {711} plane and {311} plane with a smaller area, the rugged planes from presumed {301} (or {401}–{203}) and {331} families appear. As etching continues, the {711} and ‘smaller’ {311} planes on the pyramid sidewall disappear and smooth {311} plane from the nearby convex corner starts to dominate, as shown in Figure 5b. Evolution of the similar shapes is observed in the cases of square islands with sides along <810> and <910> crystallographic directions, as shown in Figure 6. At the beginning of etching, the pyramid sidewalls will be the planes of the {811} and {911} families, respectively. Convex corners are defined by two smooth planes of {311} family in both cases, as shown in Figure 6. As in the previous case, one of the {311} planes takes over after a sufficiently long etching time. In all three cases, etched silicon structures become pyramids defined by {311} planes with convex corners undercut asymmetrically by the rugged planes of {301} (or {401}–{203}) and {331} families, as shown in Figure 5b and Figure 6. It can be noticed that planes of {711}, {811} and {911} families also consist of consecutive facets of negligible areas.

The appearance of the planes directly under the masking layer can be noticed in all figures of simulated etching profiles [13]. These planes have smaller surface areas than the dominant ones and form shapes resembling ship prows. The planes obtained in simulation are more round and the edges of the convex corners tend to soften, so it is difficult to determine the orientation of small etched surfaces. There is a good agreement between dominant crystallographic planes obtained through experiments and simulations.

The most important result is the case of square island with sides along <310> crystallographic directions. In this case, the 3D shape will not be changed during etching. The convex corner compensation is not necessary as undercutting of {301} planes is not so severe. Together with square island with sides along <110> crystallographic directions (where convex corner compensation is applied), it could be used for future designs of sensors and actuators, as shown in Figure 2c. After a sufficiently long etching time, all square islands with sides along <n10> crystallographic directions, where n>1, become silicon truncated pyramids defined by {311} planes with convex corners undercut asymmetrically by the rugged planes of {301} (or {401}–{203}) and {331} families.

### 3.2. Square Apertures in the Masking Layer with Sides Along <n10> Crystallographic Cirections

**Figure 7:**
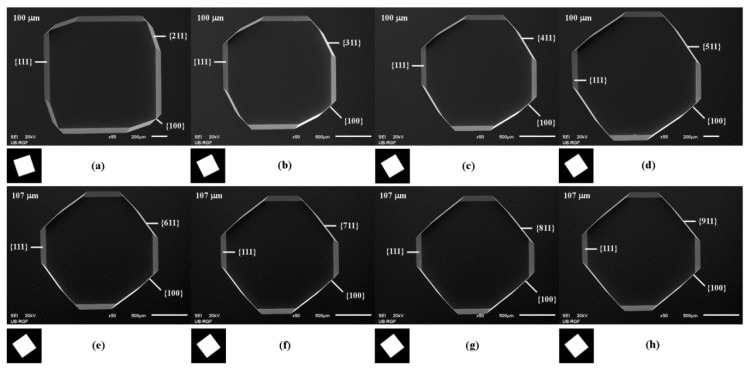
Schematic mask patterns, SEM micrographs of the etched square apertures: (**a**) <210>; (**b**) <310>; (**c**) <410>; (**d**) <510>; (**e**) <610>; (**f**) <710>; (**g**) <810>; (**h**) <910>. The etching depths were 100 and 107 µm.

3D shapes obtained by etching of square apertures in the masking layer with sides along <n10> crystallographic directions also have some similarities. All obtained cavities have a bottom that is determined by {100} plane of etched silicon substrate. Sides of squares aperture aligned to the <n10> direction allow developing of {n11} crystallographic planes at the beginning of etching. The concave corners are defined by the slowest etching planes, as shown in Figure 7.

The two most studied cavities were fabricated by etching square apertures in the masking layer with sides along <110> and <100> crystallographic directions. In the first case, sidewalls of a cavity are defined by the slowest etching planes {111}. In the second case, sidewalls of a cavity are defined by the planes of {100} and {111} families. After a sufficiently long etching time, the cavity will be changed into an inverse pyramid with the sides that are defined by {111} planes, as in the first case.

In all other cases of cavities obtained by etching of the square apertures with sides along <n10> crystallographic directions (1 < n < 10), the initial right concave corners are turned into three new concave corners, as shown in Figure 7. The first new concave corner is defined by the slowest etching planes {111} and {n11}. Appropriate <110> and <n10> crystallographic directions form angle smaller than 45° (in fact, concave angle in the masking layer larger than 145°). The second concave corner is defined by planes of {n11} and {100} families. The third concave corner is defined by the planes of {100} and {111} families. Appropriate <n10> crystallographic direction form the concave angle with <110> direction in the masking layer larger than concave angle formed by <110> and <100> crystallographic directions. After a sufficiently long etching time, cavities will have shape as in the case of etching square aperture in the masking layer with sides along <100> direction. Further etching will produce inverse pyramid with sides that are defined by {111} planes.

### 3.3. Circle Island and Aperture in the Masking Layer

**Figure 8:**
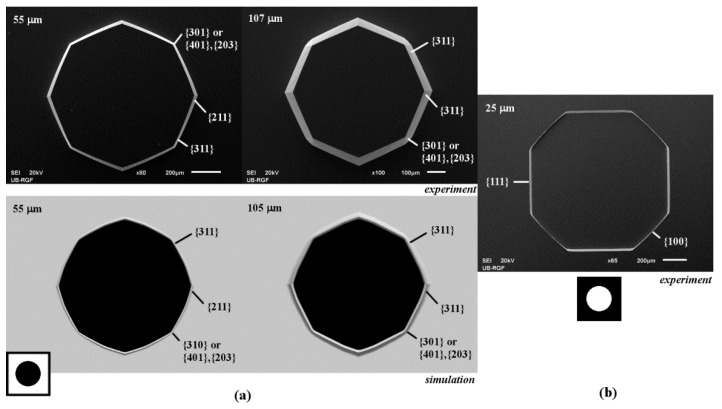
Schematic mask patterns, SEM micrographs and simulated etching profiles of the etched circle: (**a**) island; (**b**) aperture. In the experiment the depths of etching for island are 55 and 107 µm. In the experiment the etching depth for aperture was 25 µm. In the simulation the depths of etching were 55 and 105 µm.

The 3D shape obtained by etching of circle island in masking layer is similar to the shapes obtained in the case of square islands with sides along <110> and <100> directions after a sufficiently long etching time, as shown in Figure 8a. At the beginning of etching, the first type of convex corner is defined by the rugged planes of {301} family (or {401}–{203} families) and the smooth planes of {311} families, as shown in Figure 8a. The second type of convex corner is defined by two smooth planes of {211} family. As etching continues, the plane of {211} family on the sidewall of pyramid disappears and smooth plane of {311} family from the nearby convex corner takes over. Further etching will not change the silicon structure defined by these convex corners.

The cavity obtained by etching of the circle aperture in the masking layer is similar to the cavity obtained by etching of the square aperture with sides along <100> crystallographic directions. At the beginning of the etching, the transition of plane {100} to {111} at the concave corner is not so abrupt as the transition at concave corner of the square aperture, as can be observed from Figure 8b. As in other cases of apertures, after a sufficiently long etching time, the cavity will become an inverse pyramid with the sides defined by the slowest etching planes of {111} family.

### 3.4. Etch Rates of Exposed Planes

**Figure 9:**
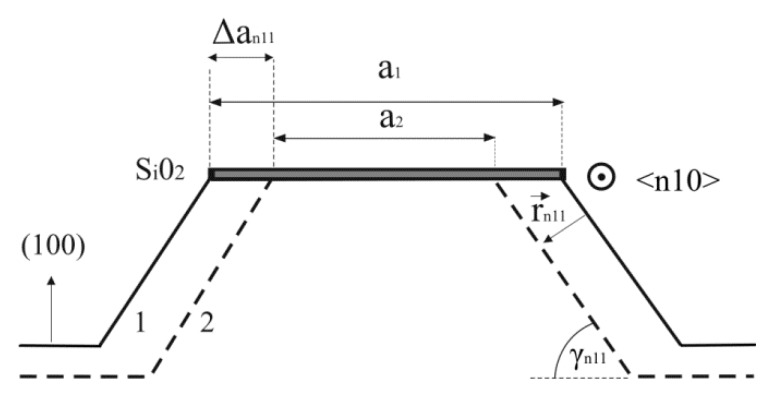
Schematic picture of cross section of the etched square island with sides along <n10> directions. *a_1_* and *a_2_* are the sides of the square in the <n10> direction for the moments *t_1_* and *t_2_*. *r_n11_* is etch rate. *γ_n11_* is the angle between {n11} and (100) planes.

Table 1 includes the etch rates for all dominant exposed crystallographic planes during etching that have not been considered in our previous work [12,13]. The etch rate of {100} plane is 0.46 µm/min. The etch rates are determined indirectly by measuring change of the square side *a*_*n*11_ of the island in the masking layer with time for both the experiment and the simulated etching profile [9], as shown in Figure 9:

(1) $$r_{n11}=\left(\Delta a_{n11}/\Delta t\right)\sin\gamma_{n11}$$

where

$$\Delta t=t_{2}-t_{1},\Delta a=\left|a_{2}-a_{1}\right|$$

and where *t_1_* and *t_2_* are two subsequent moments of the etching, *a_1_* and *a_2_* are the sides of the square in the <n10> direction for the moments *t_1_* and *t_2_* and *γ_n11_* is the angle between {n11} and (100) planes. All angles used in (1) are given in Table 1. Table 1 gives insight into derivations of average angles *γ_n11_* and etch rates which are measured in the experiments and simulations. The second column are the theoretical angles, while the third column represents the input etch rates for simulation or the numerically interpolated etch rates [13,43]. The calculated etch rates for planes in the experiments are in good agreement with the values obtained by the authors of Reference [8]. The etch rates measured in the simulations change in time and they are dependent on the surface area of crystallographic plane. After some time and sufficient surface area, the etch rate will be close to its input or numerically interpolated value. It can be concluded that all differences between 3D shapes obtained in the experiments and simulations are due to the differences of their etch rates.

## 4. Conclusions

In this paper we studied silicon etching of square and circle patterns in the masking layer when 25 wt % TMAH water solution is used at the temperature of 80 °C. Almost all crystallographic planes that appear during etching are determined. Etch rates of dominant exposed planes are calculated using the derived relation. Good agreement of experimental and simulation results has been presented. In this way, we confirm that the simulations based on the level-set model can help cost reduction when designing silicon microdevices. Analyzed behavior of the crystallographic planes that appeared during etching described in this paper contributes to a better understanding of anisotropic etching in 25 wt % TMAH water solution at the temperature of 80 °C. The 3D shape of square island in the masking layer with sides along <310> crystallographic directions will not be changed during etching and no convex corner compensation is needed. This mechanism provides advantages for controllable designs of complex silicon structures not only using the most common directions <110> and <100>. In addition, other observed effects can be used in future designs of various silicon microdevices.

## Figures and Tables

**Table 1:** The etch rates of various crystallographic Si planes in the 25 wt % TMAH water solution at the temperature of 80 °C obtained by numerical interpolation, experiment and simulation, and average angles *γ_n11_* between {n11} planes and (100) plane in the experiment and simulation obtained from normal cross-sections and their theoretical values. We used a combination of Paraview and Gimp software tools and SEM micrographs, microscope photographs and depth mesaurments to determine angles, as in References [9,26].

*Plane {n11}*	*γ*_*n*11 *theo*_ (°)	Etch Rate *r*_*n*11 *input*_ (µm/min)	*γ*_*n*11 *exp*_ (°)	Etch Rate *r*_*n*11 *exp*_ (µm/min)	*γ*_*n*11 *sim*_ (°)	Etch Rate *r*_*n*11 *sim*_ (µm/min)
*{111}*	54.7	0.02	54.2	0.02	54.7	0.03
*{211}*	65.9	0.87	66.7	0.87	65.3	0.81
*{311}*	72.5	0.93	74.2	0.93	69.8	0.88
*{411}*	76.4	0.82	78.7	0.85	76.9	0.79
*{511}*	78.9	0.75	80.9	0.81	79.4	0.74
*{611}*	80.7	0.71	81	0.73	80.8	0.70
*{711}*	82	0.67	83.1	0.69	82.1	0.66
*{811}*	82.9	0.65	83.1	0.66	82.9	0.64
*{911}*	83.7	0.63	84.1	0.63	83.8	0.62
